# ﻿On five new species of the genera *Araneus* and *Hypsosinga* (Araneae, Araneidae) from Vietnam

**DOI:** 10.3897/zookeys.1161.102375

**Published:** 2023-05-11

**Authors:** Xiaoqi Mi, Shuqiang Li, Dinh-Sac Pham

**Affiliations:** 1 College of Agriculture and Forestry Engineering and Planning, Guizhou Provincial Key Laboratory of Biodiversity Conservation and Utilization in the Fanjing Mountain Region, Tongren University, Tongren 554300, Guizhou, China Tongren University Tongren China; 2 Institute of Zoology, Chinese Academy of Sciences, Beijing 100101, China Institute of Zoology, Chinese Academy of sciences Beijing China; 3 Vietnam National Museum of Nature (VNMN), Vietnam Academy of Science and Technology (VAST), 18 Hoang Quoc Viet, Cau Giay, Hanoi, Vietnam Vietnam Academy of Science and Technology Hanoi Vietnam

**Keywords:** Arachnida, biodiversity, diagnosis, morphology, taxonomy

## Abstract

Five new species of the spider family Araneidae Clerck, 1757 from Vietnam are described: *Araneuseugenei***sp. nov.** (♂♀), *A.ethani***sp. nov.** (♀), *A.liami***sp. nov.** (♂♀), *Hypsosingaryani***sp. nov.** (♂♀), and *H.zioni***sp. nov.** (♀). Diagnostic photographs of the habitus and copulatory organs are provided. Types of the new species are deposited in the Institute of Zoology, Chinese Academy of Sciences (IZCAS) in Beijing, China.

## ﻿Introduction

A comprehensive checklist of spiders from Vietnam was first compiled by Pham et al. (2007), who listed 320 spider species in 32 families and 159 genera. The number of spider species in Vietnam was later increased to 456 species of 41 families by Ono et al. (2012), who included 23 genera and 68 species of araneids. Few studies on spiders of Vietnam were made after 2012 other than Lin et al. (2023) and Wang et al. (2023). However, the true number of Vietnam spider taxa is probably much higher than currently known.

The goal of this paper is to describe five new species collected in three national parks (Cuc Phuong, Cat Ba, and Tam Dao national parks) in northern Vietnam.

## ﻿Material and method

All specimens were collected by canopy fogging, leaf-litter sieving, or hand collecting and are preserved in 75% ethanol. Type specimens of new species are deposited in the Institute of Zoology, Chinese Academy of Sciences (**IZCAS**) in Beijing. The specimens were examined with an Olympus SZX16 stereomicroscope. The epigynes were cleared in lactic acid for examination and imaging. The left male palps were dissected in ethanol for examination, description, and imaging. Photographs of the habitus and copulatory organs were taken with a Kuy Nice digital camera mounted on an Olympus BX43 compound microscope. Compound focus images were generated using Helicon Focus v. 6.7.1.

All measurements are given in millimeters. Leg measurements are given as total length (femur, patella + tibia, metatarsus, tarsus). Abbreviations used in the text and figures are as follows: **ALE** anterior lateral eye; **AME** anterior median eye; **C** conductor; **CD** copulatory duct; **CO** copulatory opening; **E** embolus; **FD** fertilization duct; **MA** median apophysis; **MOA** median ocular area; **PLE** posterior lateral eye; **PME** posterior median eye; **Sc** scape; **Sp** spermatheca; **TA** terminal apophysis.

## ﻿Taxonomy

### ﻿Family Araneidae Clerck, 1757

#### ﻿Genus Araneus

Taxon classificationAnimaliaAraneaeAraneidae

Clerck, 1757

08C4EFAB-F6F2-58A5-B58D-C7F6E9344A3C

##### Type species.

*Araneusangulatus* Clerck, 1757.

##### Comments.

Although the three new *Araneus* species in this paper differ greatly from the type species *A.angulatus* in both somatic and copulatory organs, they are placed in *Araneus* provisionally until a phylogenetic analysis is conducted. The three new species, along with *A.bidentatus* Mi & Li, 2022, *A.bidentatoides* Mi & Li, 2022, and *A.semiorbiculatus* Mi & Li, 2022, show some common somatic characters, such as a more or less dark brown carapace, eyes with black bases, an abdomen that is longer than wide, a female abdomen with at least a pair of low humps; these characters indicate these species must be closely related, although their copulatory organs differ.

#### ﻿ Araneus eugenei sp. nov.

Taxon classificationAnimaliaAraneaeAraneidae

E47070F2-E018-5456-9278-0D145D04258D

https://zoobank.org/128B14DB-E864-4C0C-8DC3-A79F00F21227

Figs 1 , 2 , 9A–D

##### Type material.

***Holotype*** ♂ (IZCAS-Ar44127), Vietnam: Vinh Phuc Province, Tam Dao National Park (21°31.56'N, 105°33.15'E), 10.V.2005, Dinh-Sac Pham leg. ***Paratypes***: 1♂ (IZCAS-Ar44128), same locality and collector as holotype, 9.V.2005; 1 ♂ (IZCAS-Ar44129), same locality and collector as holotype, 12.V.2005; 1♀ (IZCAS-Ar44130), Ninh Binh Province, Cuc Phuong National Park (20°15.30'N, 105°42.55'E, ca 250 m), 18.VIII.2007, Dinh-Sac Pham leg.; 1♀ (IZCAS-Ar44131) Hai Phong Province, Cat Ba National Park, acacia plantation (20°47.27'N, 105°59.35'E, ca 40 m), 14.VII.2008, Dinh-Sac Pham leg.

##### Etymology.

The species name is a boy’s name from Vietnam; noun (name) in genitive case.

##### Diagnosis.

**Figure 1.**
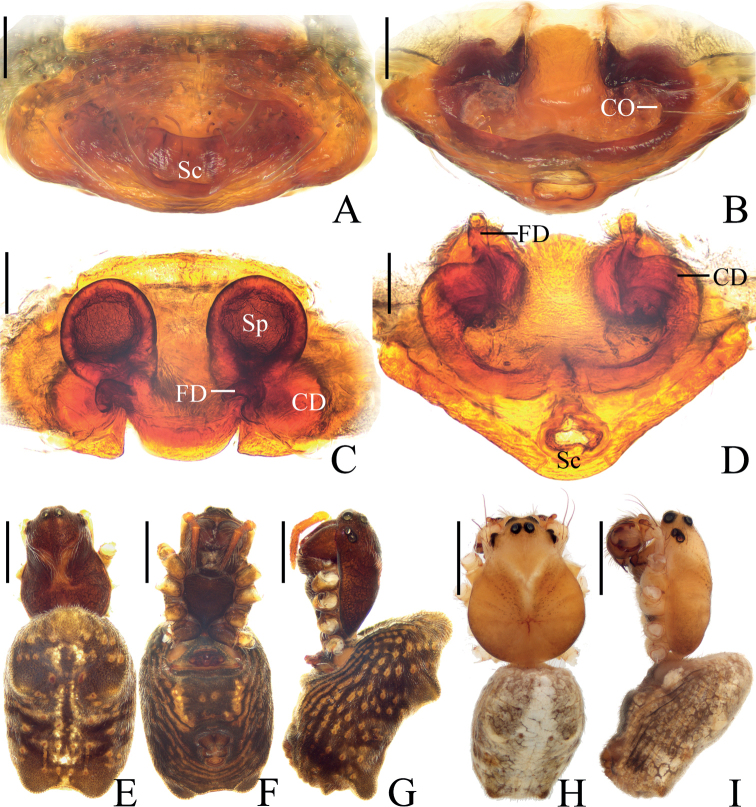
*Araneuseugenei* sp. nov. **A–G** female paratype IZCAS-Ar44131 **H, I** male holotype **A** epigyne, ventral view **B** ibid., posterior view **C** vulva, dorsal view **D** ibid., posterior view **E** habitus, dorsal view **F** ibid., ventral view **G** ibid., lateral view **H** ibid., dorsal view **I** ibid., lateral view. Scale bars: 0.1 mm (**A–D**); 1 mm (**E–I**).

**Figure 2.**
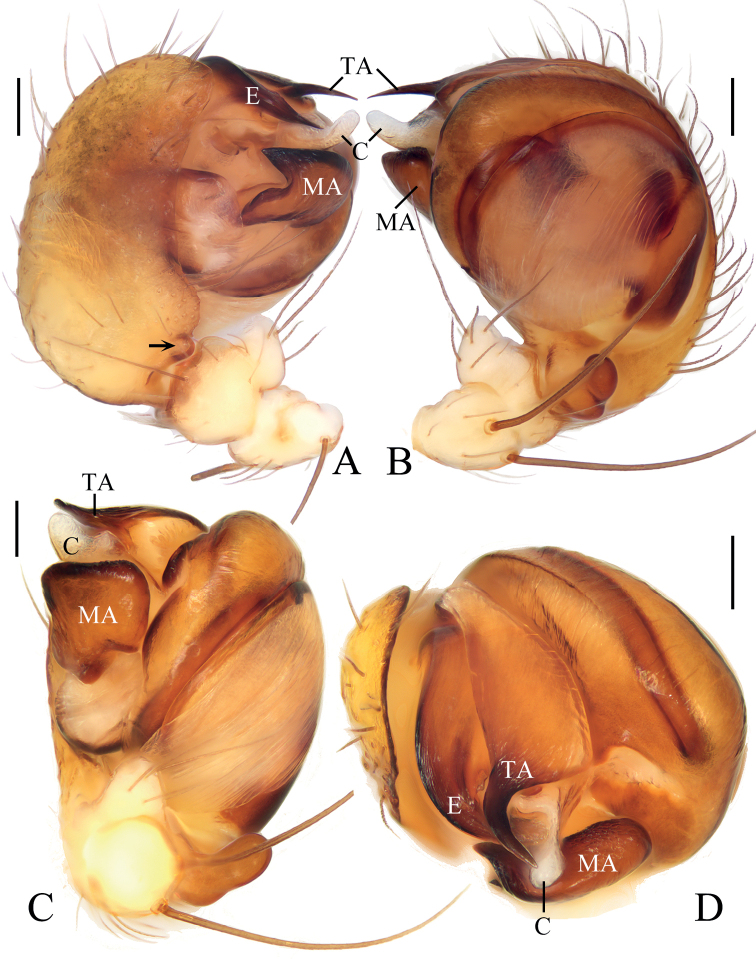
*Araneuseugenei* sp. nov., male holotype **A** left palp, prolateral view **B** ibid., retrolateral view **C** ibid., ventral view **D** ibid., apical view. Scale bars: 0.1 mm.

**Figure 3.**
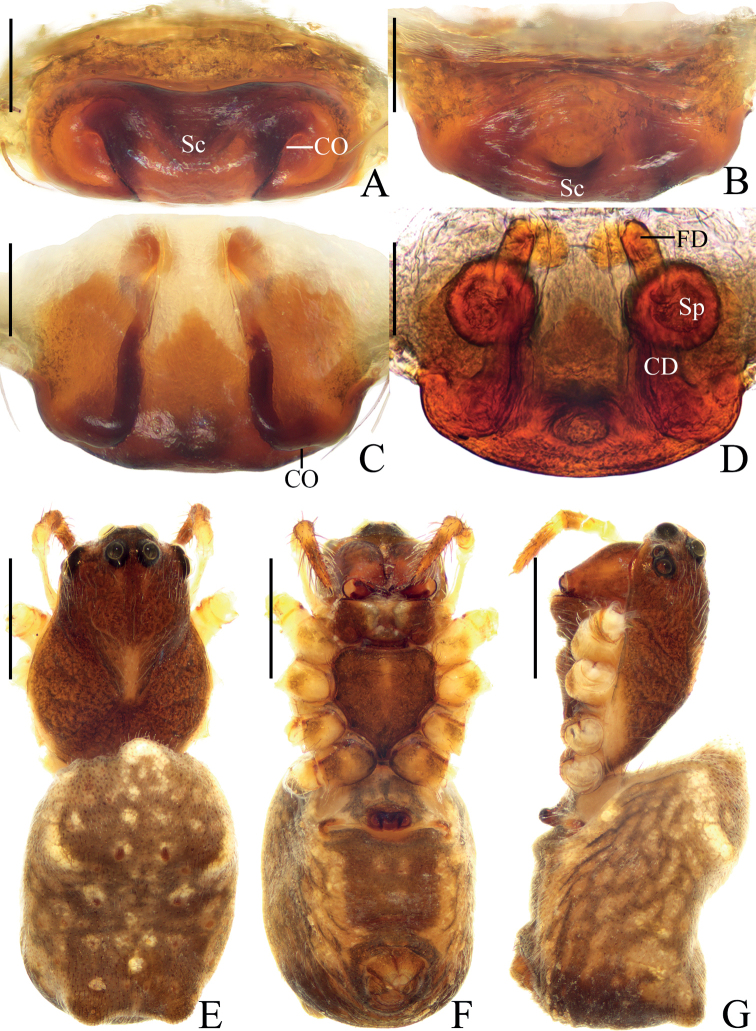
*Araneusethani* sp. nov., female holotype **A** epigyne, ventral view **B** ibid., anterior view **C** ibid., posterior view **D** vulva, posterior view **E** habitus, dorsal view **F** ibid., ventral view **G** ibid., lateral view. Scale bars: 0.1 mm (**A–D**); 1 mm (**E–G**).

**Figure 4.**
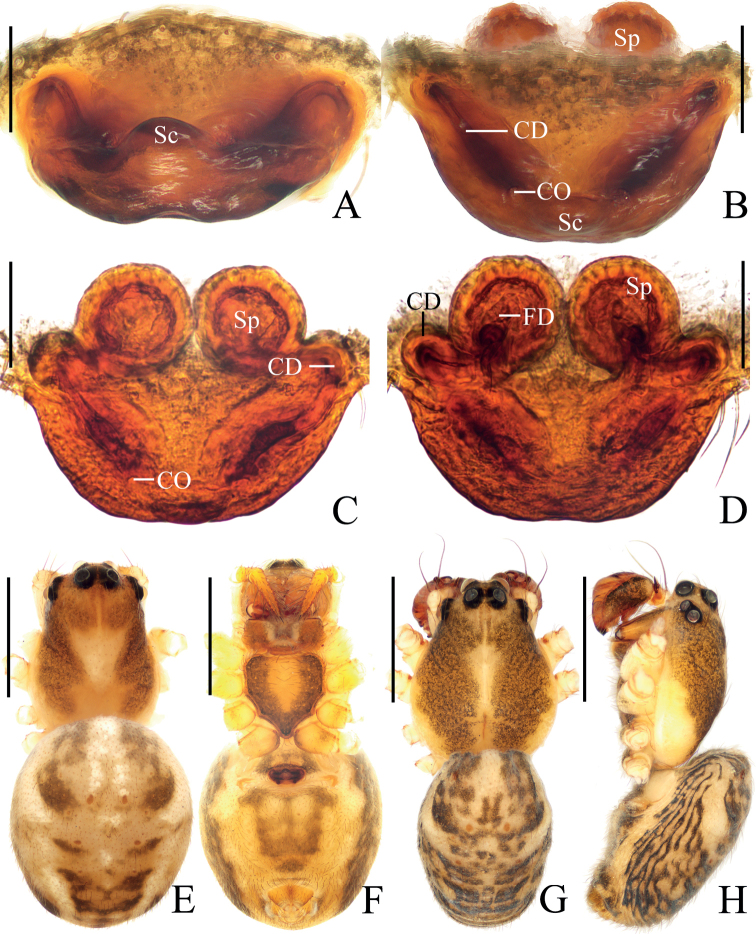
*Araneusliami* sp. nov. **A–F** female paratype IZCAS-Ar44147 **G, H** male holotype **A** epigyne, ventral view **B** ibid., anterior view **C** vulva, anterior view **D** ibid., posterior view **E** habitus, dorsal view **F** ibid., ventral view **G** ibid., dorsal view **H** ibid., lateral view. Scale bars: 0.1 mm (**A–D**); 1 mm (**E–H**).

**Figure 5.**
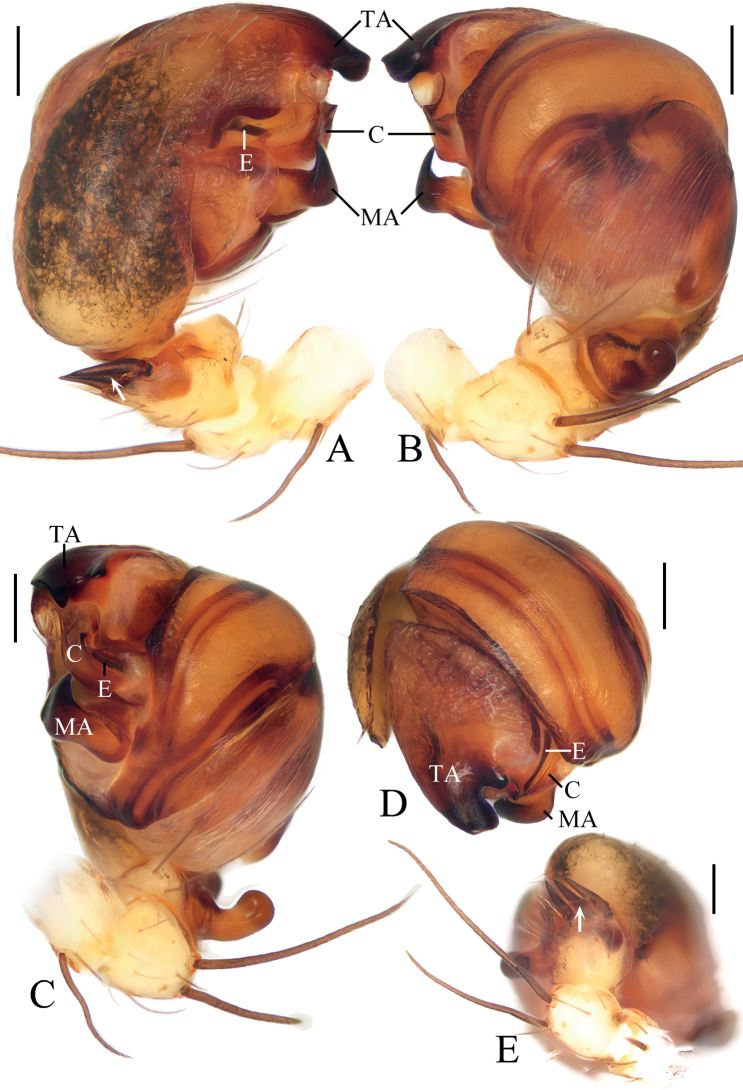
*Araneusliami* sp. nov., male holotype **A** left palp, prolateral view **B** ibid., retrolateral view **C** ibid., ventral view **D** ibid., apical view **E** tibia of left palp, dorsal view. Scale bars: 0.1 mm.

The female of the new species resembles that of *A.ethani* sp. nov. in appearance, but it can be distinguished in having 1) a triangular scape (Fig. 1B, D) vs truncated (Fig. 3B–D); 2) the copulatory openings situated on the posterior surface of the epigyne (Fig. 1B, D) vs on the ventral surface (Fig. 3A); and 3) the spermathecae separated by a distance of approximately one radius (Fig. 1C, D) vs one diameter apart (Fig. 3D). The male of the new species resembles that of *A.liami* sp. nov. in appearance, but differs in: 1) lacking heavily sclerotized denticulate protuberances on the palpal tibia (Fig. 2A–C) vs with three heavily sclerotized denticulate protuberances (Fig. 5A, E); 2) having the terminal apophysis distally pointed (Fig. 2A–D) vs bifurcated (Fig. 5C, D); 3) having the embolus tapered (Fig. 2A, D) vs thread-like (Fig. 5A, C, D); and 4) bearing a pair of low humps on the posterior part of abdomen (Fig. 1H, I) vs lacking humps (Fig. 4G, H).

##### Description.

**Male** (holotype, Figs 1H, I, 2, 9A–D). Total length 3.85. Carapace 2.00 long, 1.55 wide. Abdomen 2.45 long, 1.45 wide. Clypeus 0.10 high. Eye sizes and interdistances: AME 0.13, ALE 0.08, PME 0.10, PLE 0.08, AME–AME 0.23, AME–ALE 0.15, PME–PME 0.10, PME–PLE 0.30, MOA length 0.38, anterior width 0.45, posterior width 0.25. Leg measurements: I 6.25 (1.95, 2.20, 1.40, 0.70), II 5.50 (1.75, 1.85, 1.25, 0.65), III 3.70 (1.25, 1.20, 0.70, 0.55), IV 5.20 (1.75, 1.75, 1.05, 0.65). Carapace yellowish brown, with a V-shaped paler patch anteriorly to fovea; cervical groove slightly distinct. Chelicerae yellowish brown with four promarginal and three retromarginal teeth. Endites and labium yellowish brown, with yellow edge. Sternum yellowish brown, with gray setae. Legs yellow, with brown annuli; tibia I with 11 macrosetae; tibia II with seven macrosetae; tibia III with seven macrosetae; tibia IV with six macrosetae. Abdomen elliptical, ~1.7× longer than wide, with two pairs of very low, lateral humps; dorsal abdomen with a longitudinal patch; venter grayish brown medially and with white patches laterally. Spinnerets yellow.

***Palp*** (Fig. 2): two patellar bristles; tibia ~2× wider than long; cymbium with projection on prolateral base (see arrow in Fig. 2A); paracymbium finger-like; tegulum smoothly rounded in retrolateral; median apophysis ~1.4× wider than long, heavily sclerotized, tapered end pointed to tip of cymbium; embolus ~0.5× length of bulb diameter in prolateral view, tapered distally; conductor membranous, longer than wide; terminal apophysis about half bulb diameter width at base, tapered and curved distally.

**Female** (paratype IZCAS-Ar44131, Fig. 1A–G). Total length 4.75. Carapace 2.25 long, 1.50 wide. Abdomen 3.65 long, 2.25 wide. Clypeus 0.10 high. Eye sizes and interdistances: AME 0.15, ALE 0.08, PME 0.13, PLE 0.10, AME–AME 0.20, AME–ALE 0.18, PME–PME 0.15, PME–PLE 0.38, MOA length 0.43, anterior width 0.48, posterior width 0.38. Leg measurements: I 6.05 (1.85, 2.15, 1.35, 0.70), II 5.40 (1.65, 1.90, 1.20, 0.65), III 3.85 (1.25, 1.30, 0.75, 0.55), IV 5.70 (1.85, 2.00, 1.25, 0.60). Habitus similar to that of male, but much darker, and the two pairs of humps are more obvious.

***Epigyne*** (Fig. 1A–D): ~2.2× wider than long in ventral view, scape triangular, ~3.0× wider than long in posterior view; copulatory openings arcuated, situated on posterior surface; copulatory ducts also arcuated; spermathecae spherical, spaced by about one radius.

##### Variation.

Total length: ♂♂ 3.60–3.85; ♀♀ 4.25–4.75.

##### Distribution.

Vietnam (Vinh Phuc, Ninh Binh and Hai Phong Provinces).

#### ﻿ Araneus ethani sp. nov.

Taxon classificationAnimaliaAraneaeAraneidae

A887807C-249F-53EA-9258-3C65E28A1817

https://zoobank.org/E32071BB-3898-4353-B2AD-106D9C2EE312

Figs 3 , 9E–H

##### Type material.

***Holotype*** ♀ (IZCAS-Ar44132), Vietnam: Ninh Binh Province, Cuc Phuong National Park, disturbed forest (20°16.38'N, 105°41.10'E, ca 280 m), 3.IV.2007, Dinh-Sac Pham leg. ***Paratypes***: 1♀ (IZCAS-Ar44133), same locality and collector as holotype (20°15.30'N, 105°42.55'E, ca 250 m), 4.XII.2007; 1♀ (IZCAS-Ar44134), Hai Phong Province, Cat Ba National Park, disturbed forest (20°48.25'N, 107°00.02'E, ca 80 m), 16.VII.2008, Dinh-Sac Pham leg.

##### Etymology.

The species name is a boy’s name from Vietnam; noun (name) in genitive case.

##### Diagnosis.

The new species resembles *A.eugenei* sp. nov. in appearance but differs in having: 1) the scape truncated (Fig. 3A–D) vs triangular (Fig. 1B, D); 2) the copulatory openings situated on the ventral surface of the epigyne (Fig. 3A, C) vs on the posterior surface (Fig. 1B); and 3) the spermathecae spaced by about one diameter (Fig. 3D) vs about one radius (Fig. 1C).

##### Description.

**Female** (holotype, Figs 3, 9E–H). Total length 4.10. Carapace 2.25 long, 1.55 wide. Abdomen 2.60 long, 1.75 wide. Clypeus 0.08 high. Eye sizes and interdistances: AME 0.15, ALE 0.10, PME 0.13, PLE 0.13, AME–AME 0.20, AME–ALE 0.15, PME–PME 0.15, PME–PLE 0.30, MOA length 0.48, anterior width 0.43, posterior width 0.43. Leg measurements: I 5.80 (1.75, 2.15, 1.25, 0.65), II 5.15 (1.55, 1.85, 1.10, 0.65), III 3.90 (1.25, 1.35, 0.70, 0.60), IV 5.35 (1.70, 1.90, 1.10, 0.65). Carapace brown, with yellow anteriorly to fovea and yellow edges in thoracic region, with pale setae. Chelicerae brown with five promarginal and three retromarginal teeth. Endites and labium brown at base, paler distally. Sternum with short, longitudinal, yellow patch. Legs yellow with yellowish-brown annuli. Abdomen elliptical, ~1.25× longer than wide, pointed anteriorly and with pair of lateral humps posteriorly, covered with pale setae, dorsum grayish brown with white spots; venter brown with yellow patches. Spinnerets yellowish brown.

***Epigyne*** (Fig. 3A–D): ~2.8× wider than long in ventral view; scape truncated, ~6.0× wider than long in anterior view; copulatory openings slit-like, situated on ventral surface; copulatory ducts longer than spermatheca diameter, curved about 90°; spermathecae globular, about one diameter apart.

**Male.** Unknown.

##### Variation.

Total length: ♀♀ 3.9–4.3.

##### Distribution.

Vietnam (Ninh Binh and Hai Phong Provinces).

#### ﻿ Araneus liami sp. nov.

Taxon classificationAnimaliaAraneaeAraneidae

65D30667-8EED-542F-B2E2-83A146F740D1

https://zoobank.org/AE562B9E-1FE2-42EB-B469-7735C376C98F

Figs 4 , 5 , 9I–L

##### Type material.

***Holotype*** ♂ (IZCAS-Ar44135), Vietnam: Ninh Binh Province, Cuc Phuong National Park, disturbed forest (20°21.44'N, 105°34.21'E, ca 410 m), 5.II.2008, Dinh-Sac Pham leg. ***Paratypes***: 1♀ (IZCAS-Ar44136), same locality and collector as holotype (20°20.23'N, 105°36.28'E, ca 390 m), 2.IV.2007; 1♀ (IZCAS-Ar44137), same locality and collector as holotype (20°20.23'N, 105°36.28'E, ca 390 m), 7.V.2007; 1♀ (IZCAS-Ar44138), same locality and collector as holotype (20°21.22'N, 105°37.03'E, ca 440 m), 5.VI.2007; 1♀ (IZCAS-Ar44139), same locality and collector as holotype, 5.IX.2007; 1♀ (IZCAS-Ar44140), same locality and collector as holotype (20°21.22'N, 105°37.03'E, ca 440 m), 5.IX.2007; 1♀ (IZCAS-Ar44141), same locality and collector as holotype (20°20.23'N, 105°36.28'E, ca 390 m), 3.XII.2007; 1♂ (IZCAS-Ar44142), same locality and collector as holotype (20°20.57'N, 105°36.02'E, ca 410 m), 5.II.2008; 1♀ (IZCAS-Ar44143), Vinh Phuc Province, Tam Dao National Park (21°29.23'N, 105°37.20'E, ca 870 m), 16.V.2007, Dinh-Sac Pham leg.; 1♀ (IZCAS-Ar44144), same locality and collector as IZCAS-Ar44143 (21°29.06'N, 105°37.42'E, ca 1060 m), 16.V.2007; 1♂ (IZCAS-Ar44145), same locality and collector as IZCAS-Ar44143 (21°31.57'N, 105°33.15'E, ca 1010 m), 18.IX.2007; 1♀ (IZCAS-Ar44146), same locality and collector as IZCAS-Ar44143 (21°31.57'N, 105°33.15'E, ca 1010 m), 14.I.2008; 1♀ (IZCAS-Ar44147), same locality and collector as IZCAS-Ar44143 (21°31.50'N, 105°33.43'E, ca 1060 m), 14.I.2008.

##### Etymology.

The species name is a boy’s name from Vietnam; noun (name) in genitive case.

##### Diagnosis.

The new species resembles *A.bidentatus* Mi & Li, 2022 in appearance, but can be distinguished from it in having: 1) the copulatory openings situated on the anterior surface of the epigyne (Fig. 4B, C) vs at the lateral ends of the scape groove (Mi and Li 2022: fig. 3A, B); 2) the scape not grooved (Fig. 4A–D) vs grooved (Mi and Li 2022: fig. 3A, B); 3) the tibia of the male palp with three heavily sclerotized, denticulate protuberances (see arrows in Fig. 5A, E) vs with two protuberances (Mi and Li 2022: fig. 4A, B, E); 4) the median apophysis curved about 90° (Fig. 5A–D) vs curved about 20° (Mi and Li 2022: fig. 4A); and 5) the sternum paler in the middle (Fig. 4F) vs unicolor (Mi and Li 2022: fig. 3H).

##### Description.

**Male** (holotype, Figs 4G, H, 5, 9I–L). Total length 2.80. Carapace 1.55 long, 1.20 wide. Abdomen 1.45 long, 1.10 wide. Clypeus 0.10 high. Eye sizes and interdistances: AME 0.13, ALE 0.08, PME 0.10, PLE 0.10, AME–AME 0.15, AME–ALE 0.10, PME–PME 0.10, PME–PLE 0.18, MOA length 0.33, anterior width 0.38, posterior width 0.33. Leg measurements: I 5.30 (1.60, 1.90, 1.15, 0.65), II 4.30 (1.40, 1.45, 0.90, 0.55), III 2.90 (0.95, 0.95, 0.55, 0.45), IV 4.00 (1.30, 1.35, 0.85, 0.50). Carapace dark brown, with yellow median patches anterior to and around fovea and on lateral edges of thoracic region; cervical groove inconspicuous. Chelicerae yellowish brown, with five promarginal and three retromarginal teeth. Endites and labium yellowish brown, paler distally. Sternum dark brown with wide yellow band. Legs brown with grayish-brown annuli; tibia I with 13 macrosetae; tibia II with 10 macrosetae; tibia III with six macrosetae; tibia IV with nine macrosetae. Abdomen elliptical, ~1.3× longer than wide, covered with dark setae; dorsum yellow with grayish brown patches; venter yellow with irregular grayish brown markings. Spinnerets yellowish brown.

***Palp*** (Fig. 5): with two patellar bristles; tibia ~3.0× wider than long in retrolateral view, with three heavily sclerotized, denticulate protuberances and a short ventral projection; paracymbium finger-like; tegulum smoothly rounded in retrolateral view; median apophysis longer than wide, with pointed tip bent about 90°, distal end pointed toward the tip of cymbium in prolateral view; embolus thread-like; conductor curled, covering most of embolus in prolateral view; terminal apophysis about as long as bulb diameter, bifurcated distally.

**Female** (paratype IZCAS-Ar44147, Fig. 4A–F). Total length 3.10. Carapace 1.50 long, 1.10 wide. Abdomen 1.90 long, 1.50 wide. Clypeus 0.05 high. Eye sizes and interdistances: AME 0.13, ALE 0.08, PME 0.13, PLE 0.10, AME–AME 0.13, AME–ALE 0.13, PME–PME 0.13, PME–PLE 0.23, MOA length 0.38, anterior width 0.35, posterior width 0.35. Leg measurements: I 4.40 (1.30, 1.60, 0.95, 0.55), II 3.75 (1.15, 1.30, 0.80, 0.50), III 2.75 (0.90, 0.90, 0.50, 0.45), IV 3.90 (1.30, 1.30, 0.80, 0.50). Habitus similar to that of male, but a bit paler, yellow patches on carapace larger, and cervical groove more obvious.

***Epigyne*** (Fig. 4A–D): ~2.0× wider than long in ventral view; scape short, triangular, directed anteriorly, ~2.5× wider than long in anterior view; copulatory openings hole-shaped, located on anterior surface; copulatory ducts longer than spermatheca diameter; spermathecae globular, touching each other.

##### Variation.

Total length: ♂♂ 2.70–2.90; ♀♀ 2.90–3.45.

##### Distribution.

Vietnam (Ninh Binh and Vinh Phuc provinces).

#### ﻿Genus Hypsosinga

Taxon classificationAnimaliaAraneaeAraneidae

Ausserer, 1871

CDF24233-9391-5709-BB98-D86B3A6EFC15

Hyposinga Ausserer, 1871: 823.

##### Type species.

*Singasanguinea* C.L. Koch, 1844.

##### Comments.

The two new *Hyposinga* species in this paper differ greatly from the type species, *H.sanguinea*, in their copulatory organs. They are placed in *Hyposinga* provisionally because they show some common somatic characters, such as small total length, reflective carapace, and abdomen.

#### ﻿ Hypsosinga ryani sp. nov.

Taxon classificationAnimaliaAraneaeAraneidae

4159A7B1-F94D-5381-99AF-688CBB592ECF

https://zoobank.org/BD0AF38A-D617-4003-AA6D-37A121C784E6

Figs 6 , 7 , 10A–D

##### Type material.

***Holotype*** ♂ (IZCAS-Ar44148), Vietnam: Ha Tay Province, Ba Vi District, Tan Linh Village, 23.VII.2000, Dinh-Sac Pham leg. ***Paratype***: 1 ♀ (IZCAS-Ar44149), Cao Bang Province, Sac Ha Village, 17.VII.2000, Dinh-Sac Pham leg.

##### Etymology.

The species name is a boy’s name from Vietnam; noun (name) in genitive case.

##### Diagnosis.

**Figure 6.**
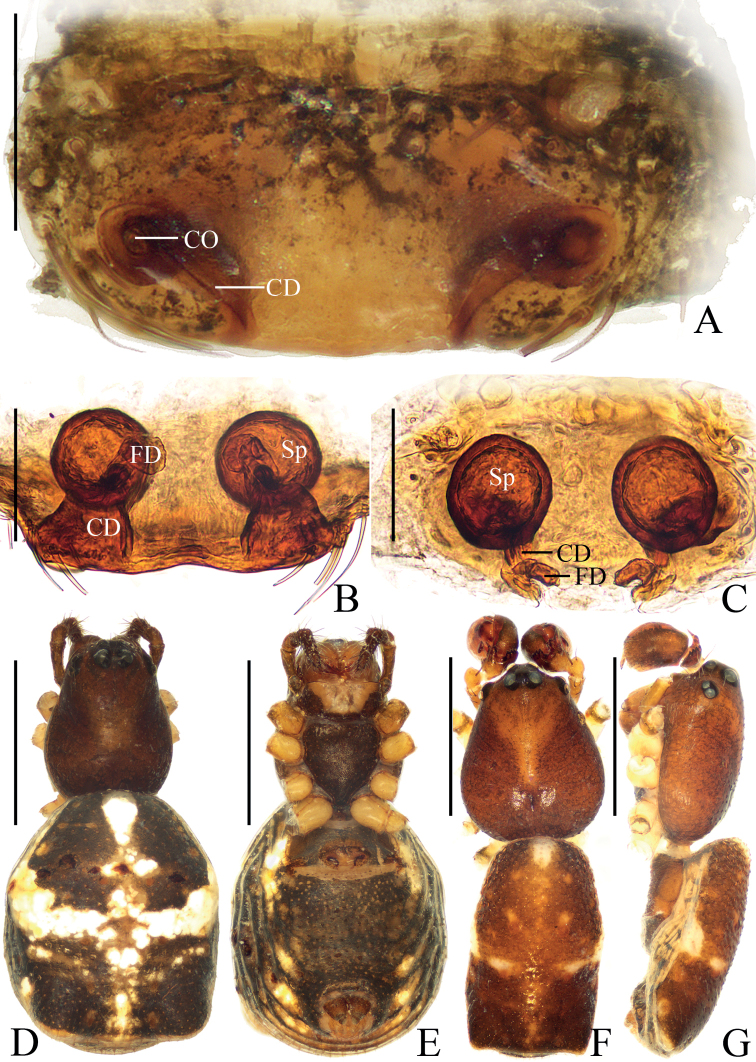
*Hypsosingaryani* sp. nov. **A–E** female paratype IZCAS-Ar44149 **F, G** male holotype **A** epigyne, ventral view **B** vulva, posterior view **C** ibid., dorsal view **D** habitus, dorsal view **E** ibid., ventral view **F** ibid., dorsal view **G** ibid., lateral view. Scale bars: 0.1 mm (**A–C**); 1 mm (**D–G**).

**Figure 7.**
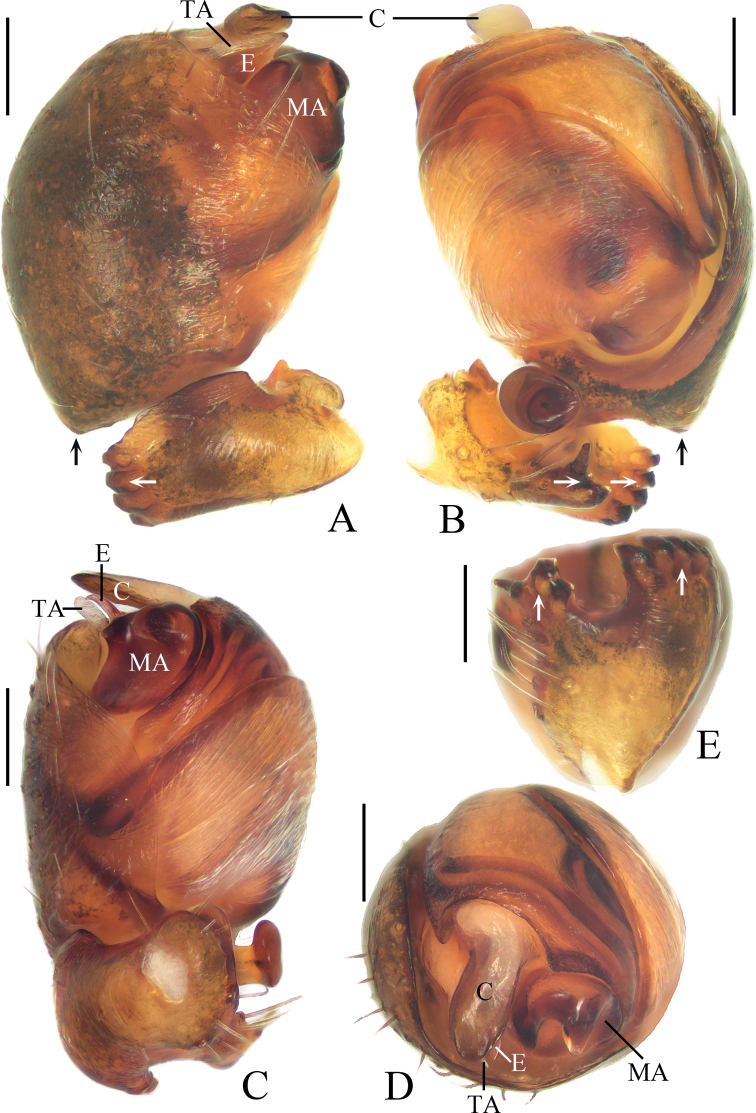
*Hypsosingaryani* sp. nov., male holotype **A** left palp, prolateral view **B** ibid., retrolateral view **C** ibid., ventral view **D** ibid., apical view **E** tibia of left palp, dorsal view. Scale bars: 0.1 mm.

The new species resembles *H.alboria* Yin, Wang, Xie & Peng, 1990 in appearance, but can be distinguished from the latter in having: 1) the epigyne lacking a septum (Fig. 6A) vs with a septum (Yin et al. 1990: fig. 182); 2) the spermathecae spaced apart by about one radius (Fig. 6B, C) vs about half of the radius (Yin et al. 1990: fig. 183); 3) the ventral surface of the epigyne smooth (Fig. 6A) vs concave (Yin et al. 1990: fig. 182); 4) the male palpal tibia palmate (Fig. 7A–C) vs not palmate; 5) the median apophysis stout (Fig. 7A, C, D) vs with a long, slender tip (Yin et al. 1990: figs 184–186); 6) the embolus short and straight (Fig. 7A, C, D) vs extremely long and curved (Yin et al. 1990: fig. 184); and 7) the tegular extension lacking (Fig. 7B–D) vs present (Yin et al. 1990: fig. 185).

##### Description.

**Male** (holotype, Figs 6F, G, 7, 10A–D). Total length 2.50. Carapace 1.15 long, 0.95 wide. Abdomen 1.40 long, 0.85 wide. Clypeus 0.20 high. Eye sizes and interdistances: AME 0.06, ALE 0.05, PME 0.10, PLE 0.06, AME–AME 0.08, AME–ALE 0.13, PME–PME 0.08, PME–PLE 0.15, MOA length 0.23, anterior width 0.20, posterior width 0.25. Leg measurements: I 3.80 (1.15, 1.30, 0.85, 0.50), II 3.45 (1.05, 1.20, 0.75, 0.45), III 2.40 (0.80, 0.75, 0.50, 0.35), IV 3.50 (1.15, 1.15, 0.75, 0.45). Carapace reddish brown, with pale stripe before fovea; cervical groove inconspicuous. Chelicerae yellowish brown; four promarginal and two retromarginal teeth. Endites and labium yellowish brown at base, paler distally. Sternum dark brown. Legs yellow to yellowish brown; leg I and II with brown annuli; tibia I with seven macrosetae; tibia II with three macrosetae; tibia III with seven macrosetae; tibia IV with two macrosetae. Abdomen ~1.6× longer than wide, with a pair of lateral humps posteriorly; dorsum reddish brown, with indistinct longitudinal pale patch and three pairs of pale spots; venter yellow to yellowish brown, with darker patches. Spinnerets yellowish brown.

***Palp*** (Fig. 7): with a single patellar bristle; tibia palmate, with bifurcated protuberance and four denticles (see white arrows in Fig. 7A, B, E); cymbium ~1.25× wider than long and covers most part of bulb in prolateral view, with dorsal protuberance at base (see black arrows in Fig. 7A, B); median apophysis elliptical at base, with two processes in apical view; tegulum smoothly rounded and lacking tegular extension in retrolateral view; embolus tapered, triangular in prolateral view, slightly curved at tip in ventral view; conductor broad at base, tongue-shaped distally in apical view; terminal apophysis membranous, narrow lamellar, about subequal in length to embolus.

**Female** (paratype IZCAS-Ar44149, Fig. 6A–E). Total length 2.55. Carapace 1.05 long, 0.80 wide. Abdomen 1.65 long, 1.35 wide. Clypeus 0.10 high. Eye sizes and interdistances: AME 0.06, ALE 0.05, PME 0.10, PLE 0.08, AME–AME 0.08, AME–ALE 0.13, PME–PME 0.08, PME–PLE 0.13, MOA length 0.20, anterior width 0.18, posterior width 0.23. Leg measurements: I 2.95 (0.90, 1.05, 0.60, 0.40), II 2.65 (0.85, 0.85, 0.60, 0.35), III 1.80 (0.60, 0.60, 0.35, 0.25), IV 2.90 (0.95, 1.00, 0.60, 0.35). Habitus similar to that of male but darker, and pale patches on dorsum of abdomen more distinct.

***Epigyne*** (Fig. 6A–C): ~2.3× wider than long in ventral view, lacking scape; copulatory openings rounded, situated at the lateral side of ventral surface; copulatory ducts about of equal length to spermatheca diameter; spermathecae globular, about one radius apart.

##### Distribution.

Vietnam (Ha Tay and Cao Bang provinces).

#### ﻿ Hypsosinga zioni sp. nov.

Taxon classificationAnimaliaAraneaeAraneidae

BFDAC6A6-0ECA-5B16-B756-EF1B916A915D

https://zoobank.org/456B12BF-5FE9-4061-B02A-14F973EB17AE

Figs 8 , 10E–H

##### Type material.

***Holotype*** ♀ (IZCAS-Ar44150), Vietnam: Cao Bang Province, Sac Ha Village,17.VII.2000, Dinh-Sac Pham leg. ***Paratypes***: 1 ♀ (IZCAS-Ar44151), Ha Giang Province, Gao Bao Village, 9.XII.2000, Dinh-Sac Pham leg.; 1 ♀ (IZCAS-Ar44152), Cao Bang Province, Sac Ha Village, 17.VII.2000, Dinh-Sac Pham leg.

##### Etymology.

The species name is a boy’s name from Vietnam; noun (name) in genitive case.

##### Diagnosis.

**Figure 8.**
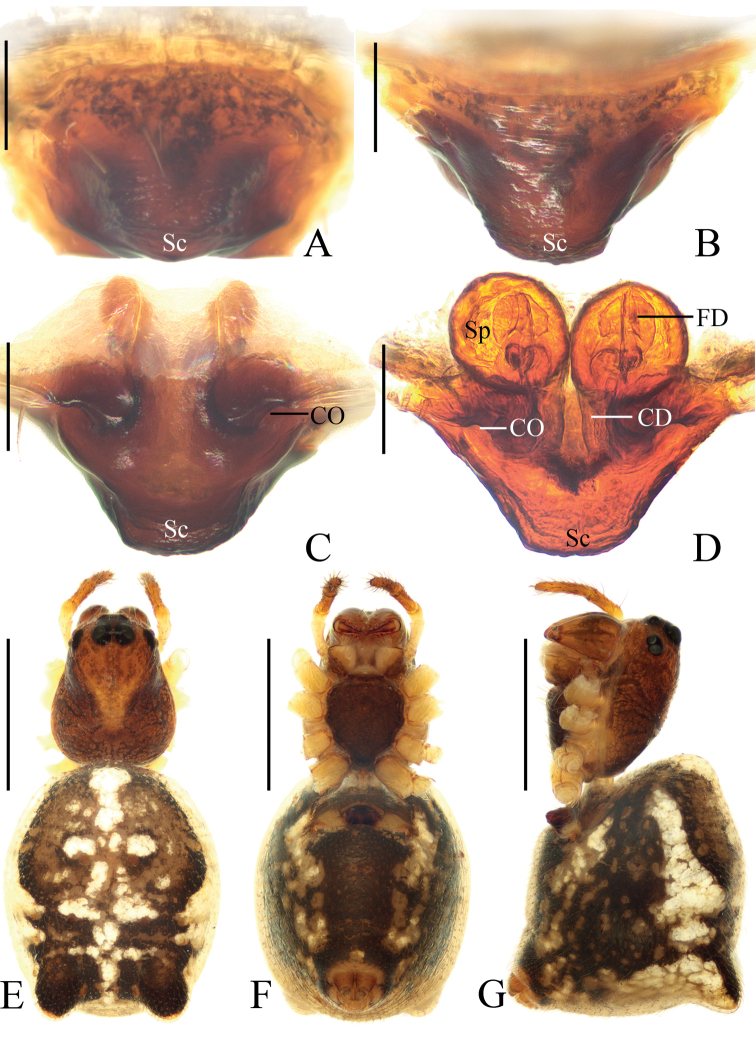
*Hypsosingazioni* sp. nov., female holotype **A** epigyne, ventral view **B** ibid., anterior view **C** ibid., posterior view **D** vulva, posterior view **E** habitus, dorsal view **F** ibid., ventral view **G** ibid., lateral view. Scale bars: 0.1 mm (**A–D**); 1 mm (**E–G**).

The new species resembles the female of *H.ryani* sp. nov. in appearance, but it can be distinguished from the latter in having: 1) the epigyne with scape (Fig. 8A–D) vs scape lacking (Fig. 6A); 2) the copulatory openings situated on the posterior surface of the epigyne (Fig. 8C, D) vs on the ventral surface (Fig. 6A); 3) the spermathecae touching each other (Fig. 8D) vs apart (Fig. 6B, C); and 4) dorsum of abdomen with three transverse bands (Fig. 8E) vs only one band (Fig. 6D).

**Figure 9.**
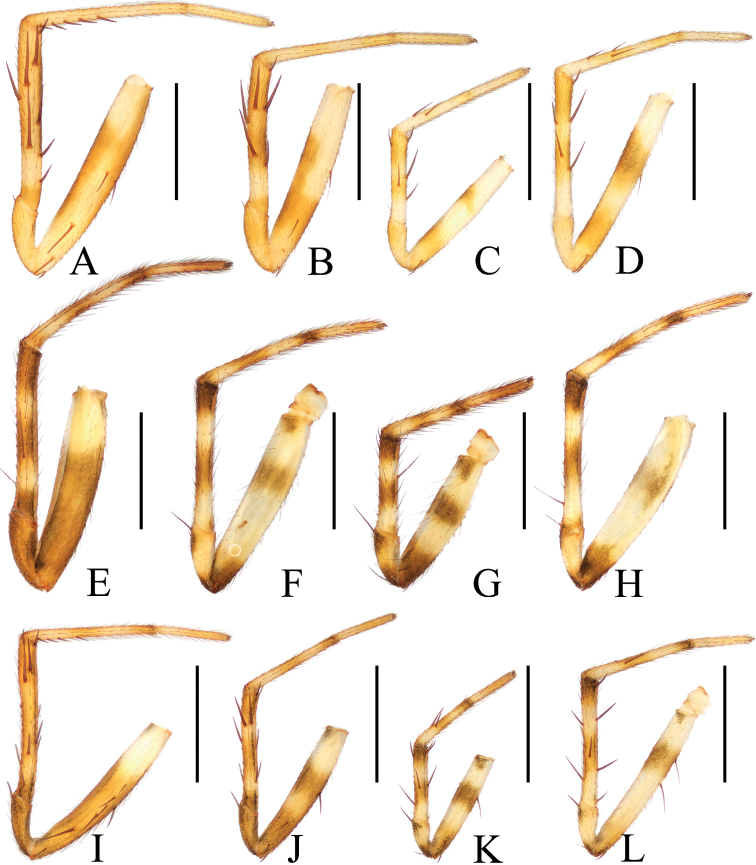
Legs of the new species, holotypes, prolateral view **A–D***Araneuseugenei* sp. nov. **E–H***Araneusethani* sp. nov. **I–L***Araneusliami* sp. nov. **A, E, I** legs I **B, F, J** legs II **C, G, K** legs III **D, H, L** legs IV. Scale bars: 1 mm.

##### Description.

**Figure 10.**
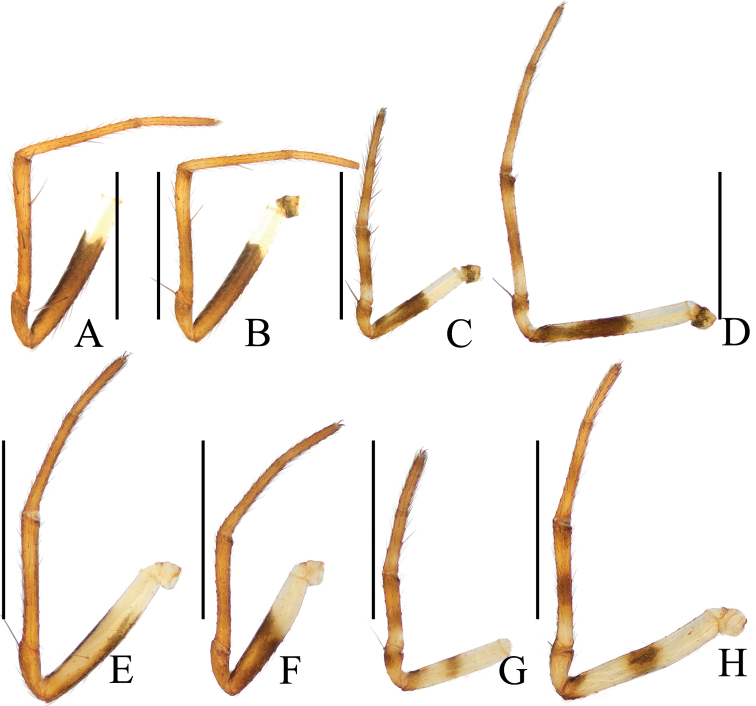
Legs of the new species, holotypes, prolateral view **A–D***Hypsosingaryani* sp. nov. **E–F***Hypsosingazioni* sp. nov. **A, E** legs I **B, F** legs II **C, G** legs III **D, H** legs IV. Scale bars: 1 mm.

**Female** (holotype, Figs 8, 10E–H). Total length 2.65. Carapace 1.10 long, 0.80 wide. Abdomen 1.70 long, 1.40 wide. Clypeus 0.13 high. Eye sizes and interdistances: AME 0.06, ALE 0.06, PME 0.10, PLE 0.08, AME–AME 0.10, AME–ALE 0.13, PME–PME 0.10, PME–PLE 0.15, MOA length 0.23, anterior width 0.20, posterior width 0.25. Leg measurements: I 3.05 (0.95, 1.05, 0.65, 0.40), II 2.70 (0.85, 0.90, 0.55, 0.40), III 1.95 (0.65, 0.65, 0.35, 0.30), IV 3.00 (1.00, 1.00, 0.60, 0.40). Carapace reddish brown; cervical groove distinct; foeva depressed. Chelicerae yellowish brown; four promarginal and two retromarginal teeth. Endites and labium dark brown at base, and paler distally. Sternum dark brown, with dark setae. Legs yellowish brown; leg III and IV with grayish brown annuli. Abdomen oval, rounded anteriorly, with a pair of lateral humps posteriorly; dorsum yellowish brown with a longitudinal median white band and three transverse white bands; also with a pair of lateral white patches; venter dark brown, with lateral pale patches. Spinnerets yellow.

***Epigyne*** (Fig. 8A–D): scape short thick, ~2.0× wider than long in posterior view; copulatory openings elliptical, situated on lateral edge of posterior surface; copulatory ducts slightly longer than spermatheca diameter, curved about 90°; spermathecae globular, touching each other.

**Male.** Unknown.

##### Variation.

Total length: ♀♀ 2.60–2.75.

##### Distribution.

Vietnam (Cao Bang and Ha Giang provinces).

## Supplementary Material

XML Treatment for Araneus

XML Treatment for Araneus eugenei

XML Treatment for Araneus ethani

XML Treatment for Araneus liami

XML Treatment for Hypsosinga

XML Treatment for Hypsosinga ryani

XML Treatment for Hypsosinga zioni
